# Cyclin B2 impairs the p53 signaling in nasopharyngeal carcinoma

**DOI:** 10.1186/s12885-023-11768-4

**Published:** 2024-01-02

**Authors:** Qinsong Liu, Yong Yuan, Xiaofen Shang, Lu Xin

**Affiliations:** Department of Otolaryngology, Qingdao Municipal Hospital, NO. 1, Shibei District, Jiaozhou Road, 266011 Qingdao, Shandong P.R. China

**Keywords:** JMJD6, CCNB2, p53 pathway, Methylation, Nasopharyngeal carcinoma

## Abstract

**Background:**

Cyclin B2 (CCNB2), a member of the cyclin family, is an oncogene in multiple cancers, including nasopharyngeal carcinoma (NPC). However, the epigenetics mechanism for CCNB2 overexpression in NPC remains unclear. This study dissects the regulatory role of CCNB2 in NPC and the molecular mechanism.

**Methods:**

Differentially methylated genes (DMG) and differentially expressed genes (DEG) were screened out in GSE52068 and GSE13597 databases, respectively, and candidate targets were identified by the Venn diagram. GO annotation and pathway enrichment analyses were performed on selected DMG and DEG, and a PPI network was constructed to pinpoint hub genes. PCR and qMSP were conducted to detect the expression and methylation of CCNB2 in cells. The siRNA targeting CCNB2 was transfected into NPC cells, and the migration, proliferation, cell cycle, epithelial-mesenchymal transition (EMT), tumorigenesis, and metastasis were examined. The upstream factor responsible for CCNB2 overexpression in NPC was explored. The p53 activity in NPC cells was assessed using western blot analysis.

**Results:**

CCNB2 showed hypomethylation and overexpression in NPC. CCNB2 silencing inhibited cell migration, proliferation, cell cycle entry, and EMT. JMJD6 was overexpressed in NPC and upregulated CCNB2 through demethylation. JMJD6 reversed the effects of CCNB2 downregulation, resulting in elevated cellular activity in vitro and tumorigenic and metastatic activities in vivo. CCNB2 blocked the p53 pathway, while the p53 pathway inhibitor reversed the effect of CCNB2 silencing to increase the activity of NPC cells.

**Conclusions:**

JMJD6 enhanced CCNB2 transcription by demethylating CCNB2, thereby repressing the p53 pathway and promoting NPC progression.

**Supplementary Information:**

The online version contains supplementary material available at 10.1186/s12885-023-11768-4.

## Background

Nasopharyngeal carcinoma (NPC) of the undifferentiated subtype is still endemic in southern China, with a peak incidence reaching 30 per 100,000 persons each year [1]. Radiotherapy remains the main treatment modality, and radiation therapy combined with chemotherapy is recommended for patients with locoregionally advanced tumors [2]. Overall prognosis has greatly improved over the past 30 years thanks to advances in management, including the advance in radiotherapy technology, the wider application of chemotherapy, and more precise disease staging, while distant failure remains an obstacle [3]. The epithelial-to-mesenchymal transition (EMT), which results in cancer metastasis, is influenced by epigenetic factors such as DNA methylation and non-coding RNAs [4]. Therefore, advances in the molecular mechanism underlying EMT might help to illuminate the pathogenesis of NPC.

Proteins in the cyclin family modulate the levels of cyclin-dependent kinases, and the cyclin B (CCNB) group stimulates the progression of the G2/M transition [5]. For one of the subfamilies, CCNB2 has been verified to be significantly associated with tumor number, tumor size, tumor thrombus, and alanine aminotransferase level in patients with hepatocellular carcinoma [6]. More importantly, gene-expression profiles in NPC in the GSE13597 dataset (an FDR *p* < 0.05 and an |log2 FC| > 1) revealed that CCNB2 was the most enriched gene and that depletion of CCNB2 stimulated apoptosis and cell cycle arrest of NPC cells [7]. However, the mechanism responsible for its upregulation in NPC has not been revealed. In the present study, with the help of GEO datasets, we also identified CCNB2 as both a significantly demethylated gene and an overexpressed gene in human NPC tissues relative to normal control. As a consequence, we postulated that the upregulation of CCNB2 in NPC was due to its demethylation status.

Methylation alterations in the promoter or first exon CpG island can result in the silencing of gene expression [8]. Moreover, jumonji domain-containing protein 6 (JMJD6), also termed PTDSR or PSR, has been described to serve as a histone arginine demethylase or lysyloxidase, hence eliciting the dynamic modulation of gene transcription [9]. Intriguingly, a positive correlation between cell cycle regulatory protein CDK4 and JMJD6 expression was identified, and mechanistic analysis indicated JMJD6 enhanced CDK4 expression in hepatocellular carcinoma by directly binding to its promoter [10]. Therefore, we hypothesized that overexpression of CCNB2 in NPC might also be attributed to JMJD6. However, this hypothesis needs to be attested. The p53 pathway is a cellular stress response network with diverse inputs and downstream outputs relevant to its role as a tumor suppressor pathway [11], and p53 is inactivated by several viral oncoproteins [12]. However, the regulation of p53 by CCNB2 has not been described. Here, we aimed to evaluate the relationship between JMJD6 and CCNB2 expression and their impact on the growth and invasiveness of NPC cell lines and nude mice via the tumor suppressor p53 pathway. The principal mechanisms were also dissected. Our study may offer a fresh perspective on the treatment of NPC.

## Materials and methods

### Bioinformatics

First, the NPC-related microarrays GSE52068 and GSE13597 were downloaded from the GEO database (https://www.ncbi.nlm.nih.gov/geo/) using “nasopharyngeal carcinoma” as the search keyword.

NPC tissues (n = 24) and normal nasal epithelial tissues (n = 24) from NPC patients (Female: 9, Male: 15) were used in the GSE52068 dataset. The age (year) distribution of the patients was: < 30, 3 (12.5%); 30–50, 18 (75%); > 50, 3 (12.5%). The tissues extracted from the patient’s genomic DNA were used to analyze genome-wide DNA methylation. Normal epithelial tissue from 3 patients with no evidence of malignancy and cancerous tissue from 10 patients with NPC were used in the GSE13597 dataset (the GSE13597 dataset does not provide the gender and age of the patients). Total RNA was extracted from the tissues, and down- or up-regulated genes in NPC were identified compared with non-malignant controls.

An analysis of variance was carried out using the limma package in the R language. For GSE52068, differentially methylated genes (DMG) were screened at *p* < 0.01. For GSE13597, differentially expressed genes (DEG) were screened at a screening threshold of *p*-value < 0.01 and |logFC| > 1.5. The ggplot package in R language was applied to plot the volcanoes of DMG and DEG. The Venn diagram online construction site (http://bioinformatics.psb.ugent.be/webtools/Venn/) was utilized to plot the Venn diagram and obtain the intersection of the two microarrays mentioned above. The ggplot package in R language was also utilized to plot the correlation and density distribution figures of gene methylation status and expression.

GO and KEGG enrichment analyses were conducted with the ClusterProfiler package (Bioconductor, Seattle, WA, USA) in the R program (version 3.6.3, NIH, Bethesda, MD, USA). Data regarding GO and KEGG pathway were downloaded from GO (http://www.bioconductor.org/packages/release/data/annotation/) and KEGG database (https://www.kegg.jp/kegg/rest/keggapi.html) [13] and visualized as cnet plots by GOplot (Bioconductor).

The Oncomine database (https://www.oncomine.org/resource/login.html), a discovery platform that collects the expression of genes related to various human diseases for public use, was utilized to obtain the expression of JMJD6 and CCNB2 in NPC. https://string-db.org/

### Cell lines and culture

Human NPC cell lines HNE3 and C666-1 were purchased from Bioshhy (Shanghai, China) and a normal immortalized nasopharyngeal epithelial cell line NP-69 was purchased from Millipore Corp (Billerica, MA, USA) . They were cultured in RPMI-1640 (Thermo Fisher Scientific Inc., Waltham, MA, USA) plus 10% FBS (Thermo Fisher).

### Cell transfection

The cells were seeded in 50-mL culture flasks and cultured in a complete medium until a 30%~50% confluence. Lipofectamine 2000 (5 µL, Thermo Fisher Scientific) was mixed with 100 µL serum-free medium and left at room temperature for 5 min. The small interfering RNAs (si) against CCNB2 (si CCNB2 1# and si CCNB2 2#) and overexpression (OE) fragment against JMJD6 (50 nmol, Promega Corporation, Madison, WI, USA) for transfection were mixed with 100 µL serum-free medium and left for 20 min at room temperature to form complexes with liposomes. The cells in the culture flasks were washed with a serum-free medium. Afterward, the complex was supplemented to a serum-free medium free of penicillin/streptomycin, mixed, and added to 50 mL culture flasks for transfection. The culture media were refreshed with complete media after a 6–8 h transfection in a 5% CO_2_ incubator at 37°C. The cells were treated with Pifithrin-α (PFTα, HY-15484, MedChemExpress, Monmouth Junction, NJ, USA), an inhibitor of the p53 pathway, at a concentration of 10 µM and transferred to a normal medium after 48 h. The siRNA sequences used are as follows: si CCNB2 1# guide sequences: 5’-AAUAUUCUCCAAAUCACUGGA-3’, passenger sequences: 5’-CAGUGAUUUGGAGAAUAUUGA-3’; si CCNB2 2# guide sequences: 5’-UCAAUAUUCUCCAAAUCACUG-3’, passenger sequences: 5’-GUGAUUUGGAGAAUAUUGACA-3’.

### RT-qPCR

Total RNA was isolated using RNAiso Plus (Takara Biotechnology Ltd., Dalian, Liaoning, China) and reversely transcribed into cDNA with the help of the PrimeScript RT Reagent kit (Takara). RT-qPCR was implemented using SYBR Premix Ex Taq (Takara) on a StepOne Real-Time PCR System (ABI Company, Oyster Bay, N.Y., USA), and the final concentrations were set for all reagents and cycling conditions in the reactions as described by the manufacturer. The obtained data were normalized to GAPDH. The results were calculated using the 2^−ΔΔCt^ method [14]. The primers for RT-qPCR were as follows: JMJD6 F: 5’-GTGCCTGGAATGCCTTAG-3’, JMJD6 R: 5’-GGTCTCCCTCTTACCGTCT-3’; CCNB2 F: 5’-ATGTGACTATTAGGCGAACT-3’, CCNB2 R: 5’-AGAGCAAGGCATCAGAAA-3’; GAPDH F: 5’-GTCTCCTCTGACTTCAACAGCG-3’; GAPDH R: 5’-ACCACCCTGTTGCTGTAGCCAA-3’.

### Transwell assay

The migration of NPC cell lines was assessed by Transwell assay. The cells (5 × 10^4^) were suspended in 200 µL serum-free medium and then loaded into uncoated Transwell chambers with 8 μm wells (Corning Glass Works, Corning, N.Y., USA). The basolateral chamber was loaded with 600 µL RPMI-1640 plus 10% FBS. The cells were incubated at 37 °C overnight. Methanol was applied to fix the cells that had migrated to the basolateral chamber, and the cells were stained with 1% crystal violet solution and counted under a light microscope (Leica Microsystems GmbH, Wetzlar, Germany) [15].

### Wound healing assays

NPC cells were seeded into 6-well plates (2 × 10^5^ cells/well) and cultured to 80–90% confluence. A sterile 10 µL pipette was used to scrape the confluent cell monolayer with equally spaced scratches. The medium was replaced with serum-free medium, and incubation was continued for 2 d. The wound width was observed and photographed under an inverted microscope (Olympus, Tokyo, Japan) at 0 and 48 h, thus calculating the 48-h migration rate of the cells.

### Cell counting kit-8 (CCK-8)

NPC cells were seeded in 96-well plates (3 × 10^3^), and incubation was continued to the indicated time points (0, 24, 48, 72 h). Then, 10 µL of CCK-8 solution (Beyotime, Shanghai, China) was added to each well for a 2-h incubation at 37 °C. The optical density (OD) value at 450 nm was measured to assess cell proliferation.

### Colony formation assay

At 48 h post-transfection, the cells were dissociated with 0.25% trypsin and resuspended in PBS. The cells (5 × 10^3^) were seeded in a 6-well plate containing 1 mL medium and incubated at 37 °C in a 5% CO_2_ incubator for 10 d until colonies were visible. The culture was terminated, and the cells were fixed with 5 mL 4% polymethanol for 15 min, and then stained with 0.5% crystal violet for 20 min. The number of colonies with over 10 cells was counted using a microscope (Leica) [16].

### Flow cytometric analysis

The cell cycle progression of NPC cells was analyzed using fluorescence-activated cell sorting (FACS) [17]. The transfected cells were plated in 96-well plates, centrifuged at 900 g for 10 min, fixed with 70% ethanol, resuspended, and stained with the mixture of 10 mg/mL PI (Sigma-Aldrich Chemical Company, St Louis, MO, USA) and 10 mg/mL RNase A (Solarbio, Beijing, China) at 4 °C for 0.5 h. Quantification of cell percentage at different cell cycles was conducted using a FACS flow cytometer (BD Biosciences, San Jose, CA, USA).

### Western blot

The cells were lysed with RIPA assay lysis buffer (Beyotime) on ice for 0.5 h. The whole cell lysates were then centrifuged at 4 °C for 20 min at 12,000 g. Protein concentrations were measured by bicinchoninic acid assay Protein Assay Kit (Beyotime). Denatured proteins were separated by 10% SDS-PAGE and transferred to polyvinylidene difluoride membranes (Millipore Corp, Billerica, MA, USA). The membranes were sealed with 5% skimmed milk or BSA for 60 min at room temperature and probed with primary antibodies to ZO-1 (33-9100, 1:1000, Thermo Fisher), E-cadherin (13-1700, 1:1200, Thermo Fisher), N-cadherin (33-3900, 1:1500, Thermo Fisher), Vimentin (ab8978, 1:1000, Abcam, Cambridge, UK), JMJD6 (sc-28,349, 1:800, Santa Cruz Biotechnology Inc., Santa Cruz, CA, USA), CCNB2 (sc-28303, 1:1300, Santa Cruz Biotechnology), p53 (sc-126, 1:2000, Santa Cruz Biotechnology), p-p53 (ab122898, 1:1000, Abcam), and GAPDH (ab8245, 1:1800, Abcam) overnight at 4 °C, and with the corresponding secondary antibody (ab205719, 1:3500, Abcam) for 60 min at room temperature. Finally, immunoblots were visualized by an ECL kit (32,209, Thermo Scientific). The intensity of the blotted bands was analyzed using ImageJ 1.8.0 (NIH, Bethesda, MA, USA).

### Chromatin immunoprecipitation (ChIP)

CCNB2 CpG island was downloaded from the MethPrimer website (http://www.urogene.org/cgi-bin/methprimer/methprimer.cgi) [18]. NPC cells were cultured to approximately 80% cell confluence and analyzed using the SimpleChIP Enzymatic Chromatin IP Kit (9003, Cell Signaling Technologies, Beverly, MA, USA) according to a previous study [19]. The cells were incubated for 10 min at room temperature with 37% formaldehyde in a culture dish, and the chromatin was obtained by ultrasonic fragmentation. Nuclease-free water, NaCl, and RNAse A were added to the chromatin samples, followed by the supplementation of 2 µL Proteinase K and a 2-h incubation at 65 °C. After purifying the DNA, the sample was diluted with ChIP Buffer and incubated with Protein G Magnetic Beads at 4 °C for 120 min. The chromatin was eluted from the antibody/Protein G Magnetic Beads using an eluent. The DNA was de-crosslinked and purified using centrifugation columns, and the amount of immunoprecipitated DNA was determined using SYBR Green PCR Master Mix and normalized against input DNA.

### qMSP

Gene methylation levels were measured using qMSP. The methylation primers for CCNB2 were downloaded from MethPrimer. A total of 1 µg DNA sample was diluted in a 1.5-mL EP tube containing 50 µL water and treated for 10 min with 5.5 µL 2 mol/L NaOH. After that, 3 µL 10 mmol/L hydroquinone and 520 µL 3 mol/L sodium bisulfite were cultured with about 50 µL mineral oil at 50 °C for 16 h. After removing the oil layer, 1 mL DNA Wizard reagent (A7280, Promega) was supplemented to the elution column, and the mixture was eluted with isopropanol and incubated with 50 µL water at 60 °C and 3 mol/L NaOH for 5 min at room temperature. The sample was then precipitated overnight with 1 µL 10 mg/mL glycoside ligand, 17 µL 10 mol/L amyl acetate, and chilled ethanol at -20 °C and centrifuged for 20 min. After being rinsed with ethanol, the samples were added to 20 µL water. The Methylamp Universal Methylated DNA Kit (P-1011-2, AmyJet Scientific Inc., Wuhan, Hubei, China) was used for PCR.

### Xenograft tumor model

Seventy-two BALB/c nude mice (4 weeks old, weight 17–22 g, sex not restricted) were purchased from Vital River (Beijing, China). Standard rodent food and water were freely accessible. The mice were allocated into 12 groups (n = 6 for per group). Next, transfected/treated NPC cells in the logarithmic growth phase were routinely dissociated by trypsin, and the cell density was adjusted to 1 × 10^6^ cells/mL. The skin of nude mice was sterilized, and the transfected/treated NPC cells were inoculated subcutaneously [20]. Tumor volumes were monitored weekly and measured using the following formula: V = π/6 (height × length × width), and the mice were euthanized by intraperitoneal injection of 1% pentobarbital sodium at 150 mg/kg at the end of week 4. The mice were judged to be successfully euthanized when they stopped breathing for 2 ~ 3 min without blinking reflex. The tumors were weighed after being removed. All studies regarding animals were accepted by the Animal Ethics Committee of Qingdao Municipal Hospital, and experiments were implemented as per the NIH Guide for the Care and Use of Laboratory Animals. The report of animal experiments is following the ARRIVE guidelines.

### In vivo metastasis assay

Seventy-two BALB/c nude mice (4 weeks old) were acquired from Vital River. The transfected NPC cells (1 × 10^6^) were resuspended in 100 mL PBS and administrated through the tail vein. The mice were euthanized by intraperitoneal injection of 1% pentobarbital sodium at 150 mg/kg after 48 d of feeding [21]. After confirming that the mice continued to have no voluntary respiration for 2 ~ 3 min and no blinking reflex, the lung tissues were removed and observed by hematoxylin-eosin (HE) staining. The lung tissues of mice were fixed in Bouin’s fixative for 6 h. The fixed lung tissues were dehydrated in a gradient concentration of alcohol, and paraffin-embedded sections about 5 μm thick were made after conventional paraffin embedding. Afterward, the tissues were dewaxed, rehydrated, and stained using a HE staining kit (Beyotime). In brief, the tissues were put into hematoxylin for 12 min, immersed in 1% hydrochloric acid alcohol for 10 s, and treated with eosin for 4 min. After the staining was completed, the lung nodule formation was observed under a microscope (Leica) after rehydration, clearance, and sealing with gum.

### Statistical analyses

Continuous variables were reported as the mean ± SD. Differences accepted for significance were set as *p* < 0.05. Each assessment was done in triplicate with 3-time repetition. All data analyses were carried out with SPSS 22.0 (IBM Corp. Armonk, N.Y., USA). An unpaired *t*-test was used to analyze the differences between the two groups. One-way or two-way ANOVA followed by Tukey’s post-hoc test was used for multiple-group comparisons. Pearson’s correlation analysis was used to detect correlations between gene expression.

## Results

### DMG and DEG in NPC

The DMG were compared between NPC tissues and normal nasopharyngeal epithelial tissues in 24 patients in the GSE52068 database. In total, 211,710 hypermethylated CpG islands and 273,867 demethylated CpG islands were found in NPC tissues (Supplementary Figure S1A). We annotated the differentially expressed CpG islands in the GSE52068 database through the GPL13534 platform and found a total of 26,982 genes with altered methylation status. Gene expression data were obtained from the GSE13597 database regarding 3 normal nasal epithelial tissues and 10 cancerous tissues from NPC patients. A total of 254 genes were upregulated, and 235 genes were significantly reduced in NPC tissues (Supplementary Figure S1B). As shown in the Venn diagram, 26,982 DMG and 424 DEG were overlapped to obtain 209 methylation-affected DEG (Supplementary Figure S1C). The specific 209 genes are shown in Supplementary Table S1. The reliability of the overlap of the two datasets was assessed according to the differential methylation changes and differential expression changes. The expression status of DEG was significantly and negatively correlated with the methylation status, in which there were more highly expressed/demethylated genes, signifying that the methylation status of these genes was opposite to the gene expression status in NPC (Supplementary Figure S1D).

### CCNB2 and the p53 pathway are the main regulators in NPC

GO annotation and KEGG enrichment analyses of these 209 genes were then performed. GO annotation showed that they were mainly involved in the biological process such regulation of chromosome separation and the regulation of mitotic sister chromatid segregation (Supplementary Figure S2A). As for cellular components, they are mainly localized in chromosomes, centromeric regions, and cyclin−dependent protein kinase holoenzyme complex (Supplementary Figure S2B). In terms of molecular functions, they mainly functioned in cyclin-dependent protein serine/threonine kinase regulator activity, cysteine-type endopeptidase inhibitor activity, and cytokine binding (Supplementary Figure S2C). The results of the KEGG pathway enrichment analysis showed that 209 intersecting genes were mainly enriched in cell cycle, human T-cell leukemia virus 1 infection, oocyte meiosis, and p53 signaling pathway (Supplementary Figure S2D). Among the four pathways only the cell cycle and p53 signaling pathway are closely related to cancer, and p53 signaling can regulate the cell cycle. Among the genes enriched in these four pathways, only CCNB2 was enriched in the four pathways. We then concluded that CCNB2 is the key gene in NPC. CCNB2 expression was significantly elevated in NPC samples from the GEO dataset GSE13597 (Supplementary Figure S2E). The Oncomine database was utilized to confirm the expression of CCNB2 in NPC, and CCNB2 was significantly overexpressed in NPC (Supplementary Figure S2F).

Through the above bioinformatics analysis, we identified the dysregulation of CCNB2 in NPC, and we subsequently validated the results of the bioinformatics analysis in vitro. The expression and methylation levels of CCNB2 were detected in cells using RT-qPCR and qMSP (Supplementary Figure S2G, H). We found that the expression of CCNB2 was significantly upregulated in NPC cell lines HNE3 and C666-1 compared with normal human nasopharyngeal epithelial cell line NP69, while the methylation level was significantly reduced.

### CCNB2 downregulation significantly inhibits NPC cell activity

**Fig. 1:**
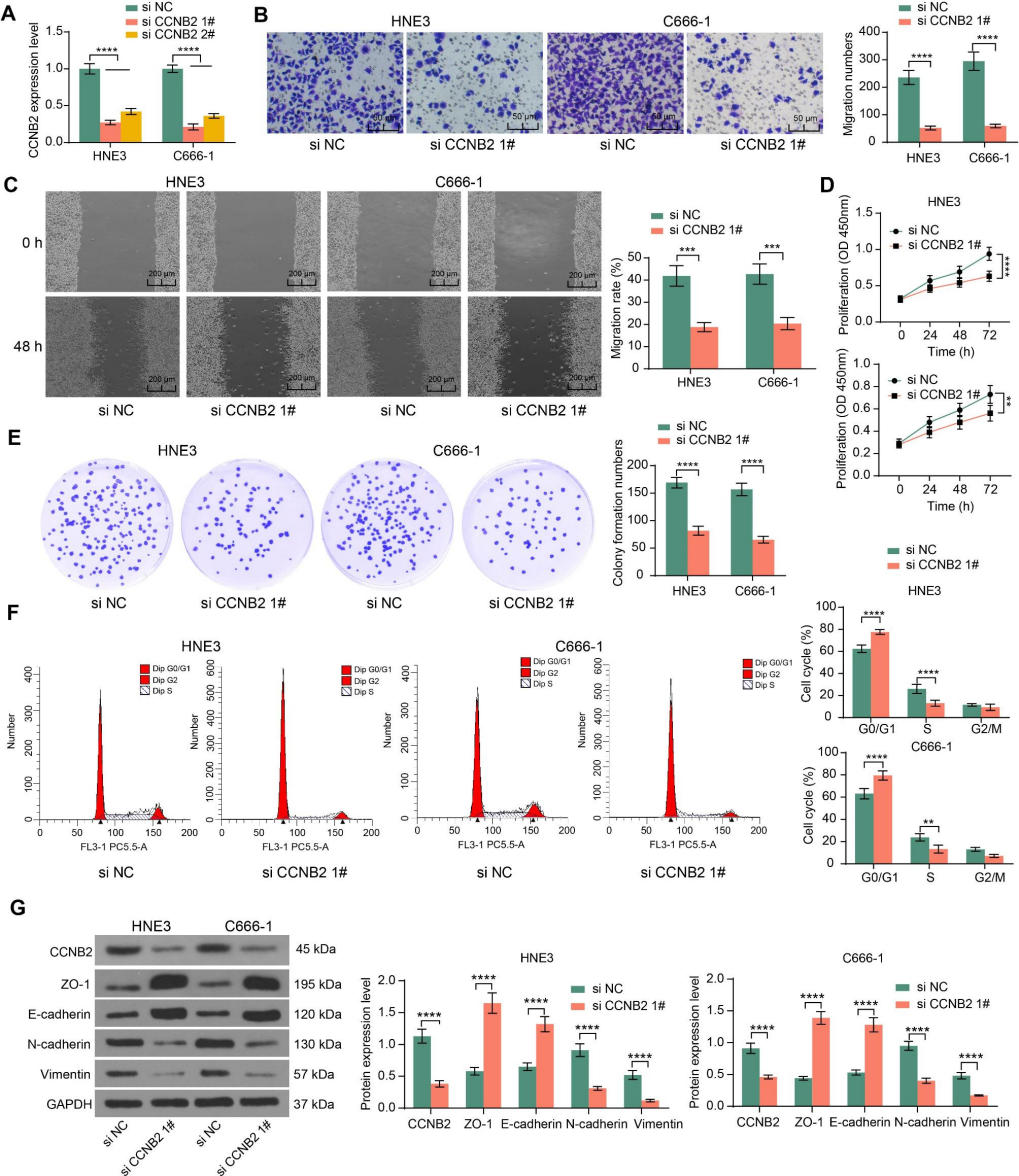
Poor expression of CCNB2 represses the proliferation, migration, and cell cycle entry of NPC cells. A, RT-qPCR was used to detect the inhibitory effect of siRNA on CCNB2 expression. The relative expression of CCNB2 was quantified according to the threshold cycle value (Ct) of qPCR by the 2^−ΔΔCt^ calculation method, and si NC was used as a control group (n = 3, analyzed by the two-way ANOVA, ****represents *p* < 0.0001). B, Transwell assay was performed to detect the migratory activity of NPC cells after inhibition of CCNB2, and the number of cells migrated to the basolateral chamber was detected after 24 h of incubation. Five nonoverlapping fields were selected to observe the cells and calculate the mean number of migrated cells (n = 3, analyzed by the two-way ANOVA, **** represents *p* < 0.0001). C, Wound healing assay was performed to detect the migration ability of cells, and wound width was measured at 0 and 48 h to calculate the 48-h migration rate of cells (n = 3, analyzed by the two-way ANOVA, *** represents *p* < 0.001). D, The OD at 450 nm was measured at 0 h, 24 h, 48 h, and 72 h of cell growth after knockdown of CCNB2, thus assessing the proliferative capacity of cells (n = 3, analyzed by the two-way ANOVA, ** represents *p* < 0.01, **** represents *p* < 0.0001). E, The effect of CCNB2 downregulation on the proliferative activity of NPC cells was detected by colony formation assays, and the number of colonies was counted under the microscope after 10 days of routine cell culture (n = 3, analyzed by the two-way ANOVA, ****represents *p* < 0.0001). F, Cells were stained with PI for 30 min and subsequently analyzed by flow cytometry for changes in the NPC cell cycle caused by inhibition of CCNB2. PI fluorescence intensity directly reflected intracellular DNA content, and cells were classified according to DNA content into G1/G0, G2/M phase, and S phase. The percentage of cells in each phase corresponding to the flow histogram was calculated by FlowJo X software (n = 3, analyzed by the two-way ANOVA, **represents *p* < 0.01, ****represents *p* < 0.0001). G, The expression of CCNB2 and EMT-related proteins after cellular downregulation of CCNB2 analyzed using western blot assays and ImageJ software (normalized to GAPDH) (n = 3, analyzed by the two-way ANOVA, ****represents *p* < 0.0001). Data are represented as mean ± SD.

SiRNA targeting CCNB2 (si CCNB2 1# and si CCNB2 2#) was transfected into NPC cells to inhibit the intracellular CCNB2 activity, and RT-qPCR showed a substantial decrease in CCNB2 expression after transfection. The si CCNB2 1# (named si CCNB2) with better transfection efficiency was selected for subsequent experimental analysis (Fig. 1A). In Transwell and wound healing assays, inhibition of CCNB2 significantly reduced the migration of HNE3 and C666-1 cell lines (Fig. 1B, C). CCK-8 and colony formation analysis showed that CCNB2 inhibition significantly attenuated the proliferation of HNE3 and C666-1 cells (Fig. 1D, E). Detection of the cell cycle revealed that poor expression of CCNB2 caused cell cycle arrest and reduced mitotic activity in NPC cells (Fig. 1F). We further tried to explore whether CCNB2 was related to EMT in NPC cells by using western blot analysis. Increased E-cadherin and ZO-1 and loss of N-cadherin and Vimentin were observed in HNE3 and C666-1 cells with poor CCNB2 expression (Fig. 1G). Thus, the results of these experiments suggest that the depletion of CCNB2 in NPC cells inhibited cell activity and EMT.

### JMJD6 regulates the methylation of CCNB2

**Fig. 2:**
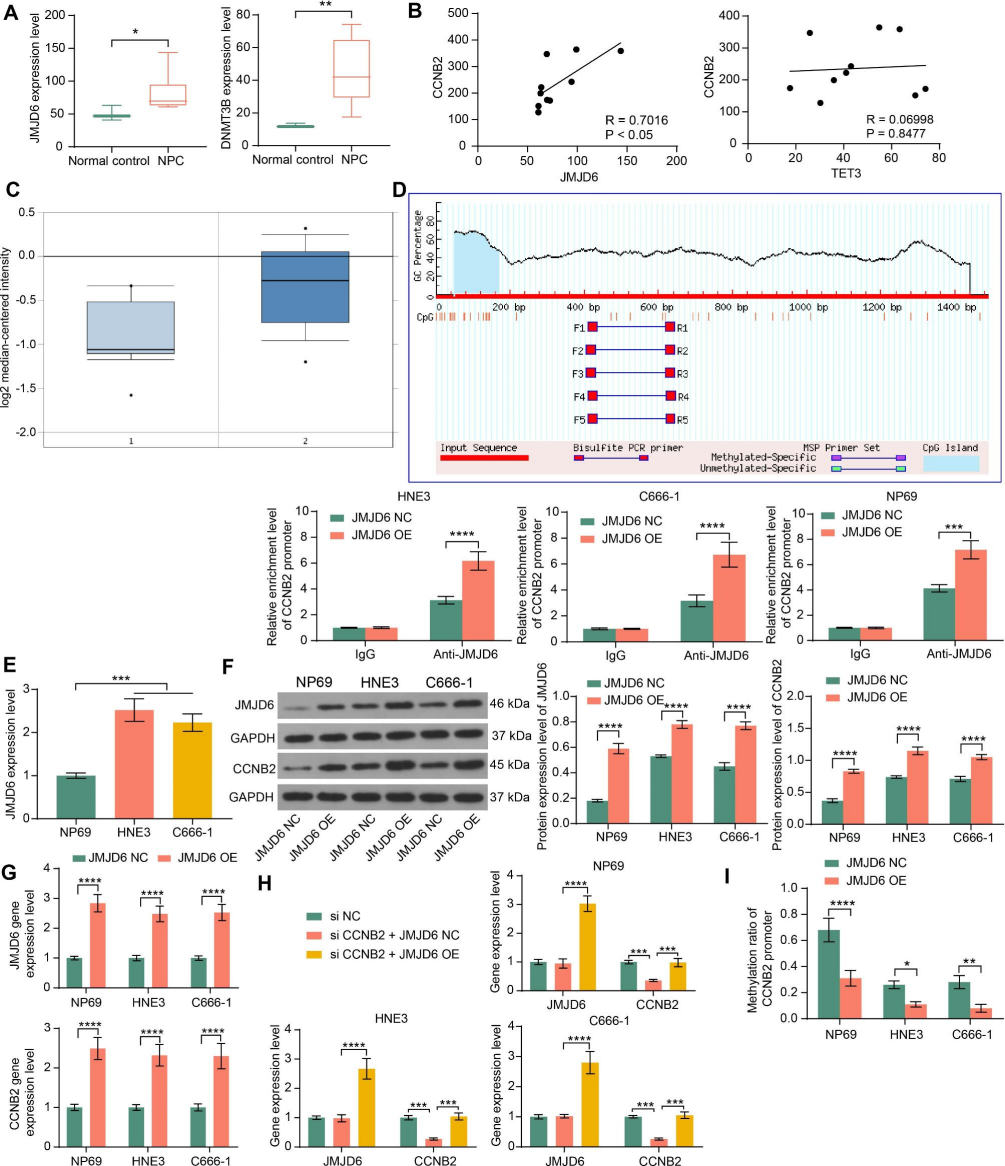
JMJD6 is an epigenetic modifier of CCNB2 in NPC. A, JMJD6 and TET3 expression in NPC samples (n = 10) and control samples (n = 3) from GEO dataset GSE13597 (analyzed by the Mann Whitney test, *represents *p* < 0.05, **represents *p* < 0.01). B, The correlation between CCNB2 and JMJD6 or TET3 expression in 10 NPC patients from the GSE13597 dataset (n = 10, analyzed by Pearson’s correlation analysis). C, The expression of JMJD6 in NPC was detected in the Oncomine database, where 1 is normal nasal tissues and 2 is NPC tissues (*p*-value = 1.03E-4, FC value = 1.533). D, The binding of JMJD6 and the CCNB2 promoter in NP69 and NPC cells was examined by ChIP assay. The relative content of the CCNB2 promoter was quantified in the immunoprecipitation of the promoter fragment pulled down by JMJD6 antibody or isotype control IgG, based on the threshold cycle value (Ct) of qPCR using the 2^−ΔΔCt^ calculation method (n = 3, analyzed by the one-way ANOVA, ***represents *p* < 0.001, ****represents *p* < 0.0001). E, RT-qPCR was performed to detect the expression of JMJD6 in NPC cell lines HNE3 and C666-1 compared to normal human nasopharyngeal epithelial cell line NP69 based on the threshold cycle value (Ct) of qPCR using the 2^−ΔΔCt^ calculation method, and NP69 was used as a control group (n = 3, analyzed by the one-way ANOVA, ***represents *p* < 0.001). F, JMJD6 and CCNB2 protein expression in cells overexpressing JMJD6 were examined using western blot. The grayscale values of the target bands (JDJD6 or CCNB2) were detected by ImageJ software and then normalized using the grayscale values of the internal reference band GAPDH (n = 3, analyzed by the one-way ANOVA, ****represents *p* < 0.0001) G, RT-qPCR was performed to detect the effect of overexpression of JMJD6 on CCNB2 expression in cells. The relative expression of the gene was quantified according to the threshold cycle value (Ct) of qPCR using the 2^−ΔΔCt^ calculation method, and JMJD6 NC was used as a control group (n = 3, analyzed by the two-way ANOVA, ****represents *p* < 0.0001). H, RT-qPCR was performed to detect the gene expression of CCNB2 or JMJD6 in cells, and the relative expression of genes was quantified according to the threshold cycle value (Ct) of qPCR by the 2^−ΔΔCt^ calculation method, si NC was used as a control group (n = 3, analyzed by the one-way ANOVA, ***represents *p* < 0.001, ****represents *p* < 0.0001). I, qMSP assays were performed to detect the effect of overexpression of JMJD6 on the methylation level of the CCNB2 promoter in cells, and the threshold cycle value of methylation qPCR/threshold cycle value of non-methylation qPCR was used to calculate the methylation level of CCNB2 promoter in each group of cells (n = 3, analyzed by the one-way ANOVA, *represents *p* < 0.05, **represents *p* < 0.01, ****represents *p* < 0.0001) Data are represented as mean ± SD.

Since CCNB2 is hypomethylated in NPC, we looked for the expression of methylation and demethylation enzymes in the GSE13597 database. Both JmjC domain-containing histone demethylases (part of the JMJD family) and the ten-eleven translocation (TET) family of 5mC DNA hydroxylases can lead to activation of oncogenic pathways and inhibition of histone and DNA demethylation [22]. JMJD6 and TET3 were differentially expressed in NPC (Fig. 2A). The correlation of their expression with CCNB2 in the database was then analyzed, and only JMJD6 was correlated with CCNB2 in NPC (Fig. 2B). We then surmised that JMJD6 regulates the methylation status of CCNB2, and noted that JMJD6 was significantly upregulated in NPC through the Oncomine database (Fig. 2C).

After obtaining the promoter sequence of the CpG island of CCNB2, the enrichment of JMJD6 on the CCNB2 promoter in cells was examined. JMJD6 protein could bind to the CCNB2 promoter and thus pull down the CCNB2 promoter fragment, and the promoter fragment pulled down by JMJD6 was also increased after overexpression of JMJD6 (Fig. 2D). As revealed by RT-qPCR, the mRNA expression of JMJD6 was significantly higher in NPC cells compared to NP69 cells (Fig. 2E). In addition, we observed a significant upregulation of CCNB2 mRNA and protein expression in NPC cells following JMJD6 overexpression (Fig. 2F, G). The regulatory effect of JMJD6 on CCNB2 was explored by upregulating JMJD6 in NPC cells with si CCNB2, and it was found that JMJD6 promoted the expression of CCNB2 (Fig. 2H). Moreover, the methylation of the CCNB2 promoter was significantly decreased in response to JMJD6 overexpression (Fig. 2I). We thus concluded that JMJD6 exerts a demethylase role in NPC cells to activate CCNB2 expression in NPC.

### JMJD6 impairs the p53 pathway via activating CCNB2

**Fig. 3:**
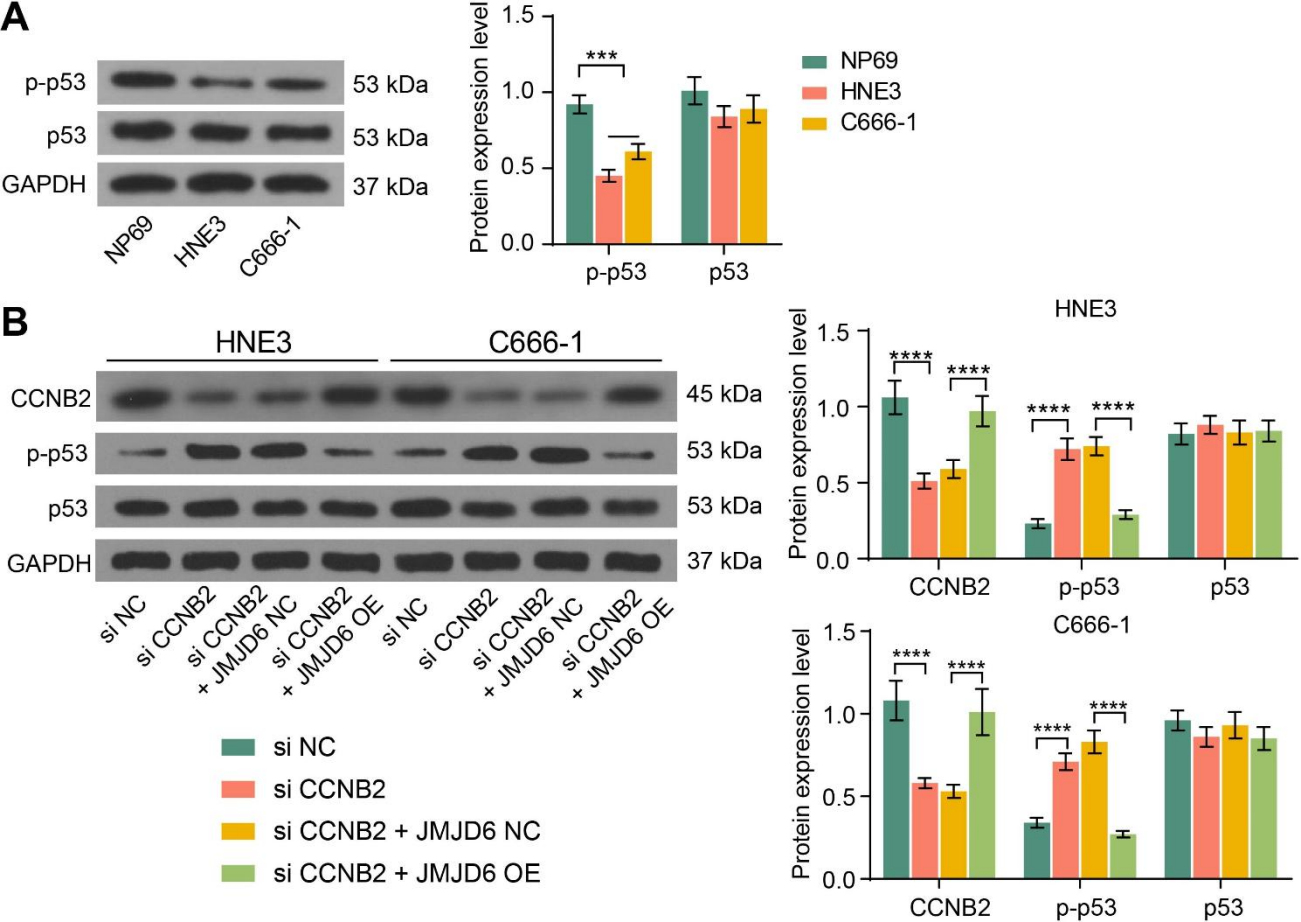
The p53 pathway is an effector pathway of the JMJD6/CCNB2 axis in NPC. A, The expression of p-p53 and p53 proteins in normal human nasopharyngeal epithelial cell line NP69, and NPC cell lines HNE3 and C666-1 using western blot assays. The grayscale values of the target bands (p-p53 or p53) were measured by ImageJ software and normalized to GAPDH (n = 3, analyzed by the two-way ANOVA, ***represents *p* < 0.001). B, The expression of CCNB2, p-p53, and p53 proteins in cells after transfection, and the grayscale values of the target bands (CCNB2, p-p53, or p53) were detected by ImageJ software before being normalized using the grayscale values of the internal reference band GAPDH (n = 3, analyzed by the two-way ANOVA, ****represents *p* < 0.0001). Data are represented as mean ± SD.

During KEGG analysis, CCNB2 was revealed to be enriched in the p53 pathway. Therefore, we examined the activity of the p53 pathway in cells using western blot and found that the p53 pathway activity was appreciably reduced in NPC cells (Fig. 3A). We found that CCNB2 downregulation significantly activated the p53 pathway, while JMJD6 upregulation suppressed p53 pathway activity in the presence of si CCNB2 (Fig. 3B).

### JMJD6 overexpression or p53 inhibitor promotes NPC cell activity in vitro

**Fig. 4:**
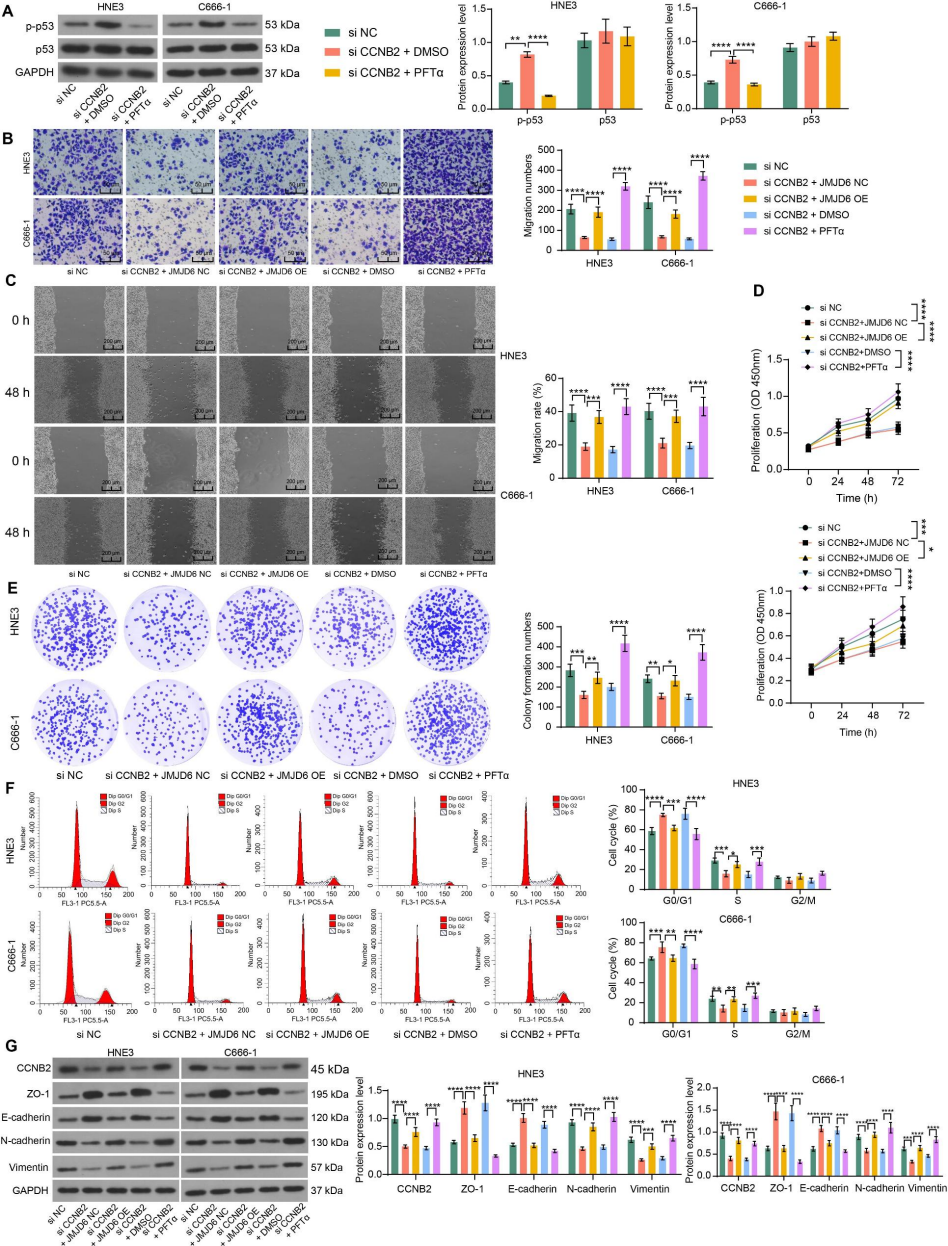
JMJD6 overexpression or impairment of the p53 pathway potentiates NPC cell proliferation and migration. A, The expression of p-p53 and p53 proteins in cells after PFTα treatment, and the grayscale values of the target bands (p-p53 or p53) were detected by ImageJ software before being normalized using the grayscale values of the internal reference band GAPDH (n = 3, analyzed by the two-way ANOVA, **represents *p* < 0.01, ****represents *p* < 0.0001). B, The cell migration was assessed by Transwell assay, and the number of cells migrated to the basolateral chamber was detected after 24 h of incubation. Five nonoverlapping fields were selected to observe the cells and calculate the mean number of migrated cells (n = 3, analyzed by the two-way ANOVA, **** represents *p* < 0.0001). C, Wound healing assay was performed to detect the migration ability of cells and wound width was measured at 0 and 48 h to calculate the 48-h migration rate of cells (n = 3, analyzed by the two-way ANOVA, ***represents *p* < 0.001, ****represents *p* < 0.0001). D, The OD at 450 nm was measured at 0 h, 24 h, 48 h, and 72 h of cell growth, thus assessing the proliferative capacity of cells (n = 3, analyzed by the two-way ANOVA, *represents *p* < 0.05, ***represents *p* < 0.001, ****represents *p* < 0.0001). E, The effect of PFTα treatment on the proliferative activity of NPC cells was detected by colony formation assays, and the number of colonies was counted under the microscope after 10 days of routine cell culture (n = 3, analyzed by the two-way ANOVA, *represents *p* < 0.05, **represents *p* < 0.01, ***represents *p* < 0.001, ****represents *p* < 0.0001). F, Cells were stained with PI for 30 min and subsequently analyzed by flow cytometry for changes in the NPC cell cycle caused by inhibition of CCNB2. PI fluorescence intensity directly reflected intracellular DNA content, and cells were classified according to DNA content into G1/G0, G2/M phase, and S phase. The percentage of cells in each phase corresponding to the flow histogram was calculated by FlowJo X software (n = 3, analyzed by the two-way ANOVA, *represents *p* < 0.05, **represents *p* < 0.01, ***represents *p* < 0.001, ****represents *p* < 0.0001). G, The expression of CCNB2 and EMT-related protein after PFTα treatment analyzed using western blot assays and ImageJ software (normalized to GAPDH) (n = 3, analyzed by the two-way ANOVA, ***represents *p* < 0.001, ****represents *p* < 0.0001). Data are represented as mean ± SD.

Pifithrin-α (PFTα) was used to inhibit the p53 pathway activity in NPC cells poorly expressing CCNB2 (Fig. 4A). The changes in cell migration after treatment were examined using Transwell and wound healing assays. Both JMJD6 and PFTα significantly inhibited the effects of si CCNB2 and increased cell migration activity (Fig. 4B, C). Meanwhile, JMJD6 and PFTα reversed the impact of si CCNB2 and enhanced cell proliferation and colony formation (Fig. 4D, E). JMJD6 and PFTα also reversed the effects of CCNB2 low expression, resulting in a significantly higher proportion of cells in the S phase (Fig. 4F). Moreover, western blot assays were conducted to examine the EMT-related proteins. JMJD6 and PFTα treatment reduced ZO-1 and E-cadherin expression and enhanced CCNB2, N-cadherin, and Vimentin expression in NPC cells (Fig. 4G). PFTα-mediated inhibition of the p53 pathway also led to the restoration of CCNB2 expression, suggesting that the inhibitory effects of CCNB2 and p53 are reciprocal.

### JMJD6 overexpression or p53 inhibitor promotes NPC cell activity in vivo

**Fig. 5:**
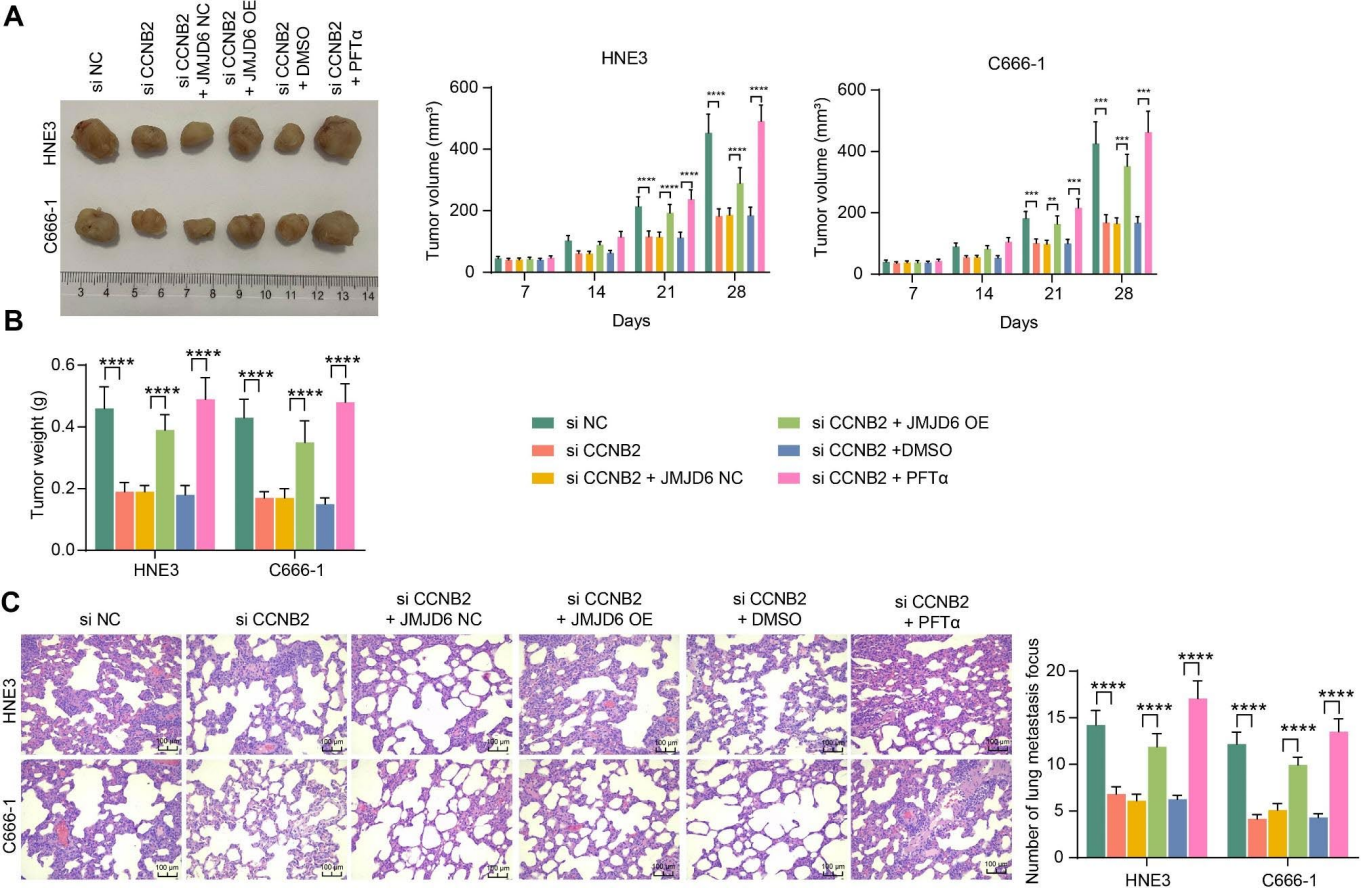
JMJD6 overexpression or impairment of the p53 pathway potentiates tumor growth and metastases in vivo. A, NPC cells were injected subcutaneously into mice to detect tumor formation in mice and tumor volumes were assessed at days 7, 14, 21, and 28 after tumor injection. The mice were euthanized after 28 days, and xenograft tumor tissues were harvested (n = 6, analyzed by the two-way ANOVA, **represents *p* < 0.01, ***represents *p* < 0.001, ****represents *p* < 0.0001). B, Subcutaneous xenograft tumor weights (n = 6, analyzed by the two-way ANOVA, ****represents *p* < 0.0001). C, NPC cells were injected into the mice through the tail vein and euthanized after 48 days. Lung tissues were collected, and the number of metastasis focus was observed by HE staining, and three nonoverlapping fields of view were selected for each section to calculate the mean number of metastasis focus (n = 6, analyzed by the two-way ANOVA, ****represents *p* < 0.0001)

NPC cells with stable transfection were delivered into nude mice to test tumor growth, and the size of the tumors generated in the nude mice was examined. It was found that CCNB2 knockdown appreciably constrained the tumorigenic activity of the cells, while JMJD6 and PFTα reversed the effect of CCNB2 to increase the tumor size in the mice (Fig. 5A). The tumor weight assay also suggests an interaction between JMJD6/CCNB2/p53 pathway (Fig. 5B). NPC cells were also administrated into the tail vein of mice to detect lung metastases. It was found that the decrease of CCNB2 reduced the metastatic activity of the cells, whereas JMJD6 and PFTα expedited the lung metastasis of the NPC cells (Fig. 5C).

## Discussion

NPC, an Epstein-Barr virus-associated cancer arising from the nasopharynx, shows distinctive characteristics concerning ethnic and geographic distribution, including higher prevalence in Southeast Asia and South China [23]. Metastasis is associated with a somber prognosis and death of cancer, and the acquisition of metastatic potential in NPC cells expedites the process of EMT [24]. Here, we established that CCNB2 was considerably upregulated in NPC. Loss-of-function studies indicated that depletion of CCNB2 repressed the proliferation, EMT, and metastases in NPC. Mechanism studies revealed that CCNB2, regulated by JMJD6, expedited NPC development by blocking the p53 signaling.

GEO analysis using two databases identified CCNB2 as one of the most overexpressed and hypomethylated genes in NPC. GO and KEGG enrichment analyses revealed the p53 signaling as the downstream effector of CCNB2. Similar to our screening process, Espinosa et al. established that CCNB2 was one of the 6 most upregulated genes between 43 human papillomavirus 16-positive cervical cancers and 12 healthy cervical epitheliums using microarrays [25]. It was also reported by Takashima et al. that the 5-year overall survival of patients with lung adenocarcinoma poorly expressing CCNB2 was significantly better than that of patients overexpressing CCNB2 [26]. Consistently, high expression of CCNB2 at the protein level was positively related to the status of differentiation, lymph node metastases, distant metastases, and clinical staging of Chinese patients with non-small cell lung cancer [27]. Functionally, the downregulation of CCNB2 hampered the proliferation, invasiveness, and cell cycle entry of lung adenocarcinoma cells [28]. Here, we substantiated the anti-proliferatory and anti-migratory properties of si CCNB2 *in vitro.* More importantly, the gain of E-cadherin and ZO-1 and the loss of N-cadherin and Vimentin were observed after the knockdown of CCNB2. These observations were novel, to the best of our knowledge. Moreover, even though the tumor-supporting effects of CCNB2 have been verified in NPC, the underlying mechanism remains largely unclear. Here, the fact that demethylation of CCNB2 caused its overexpression in NPC has been proposed and validated.

Considering multiple methylation-related enzymes were differentially expressed in NPC in the GSE13597 database. Only JMJD6 showed the closest correlation with CCNB2 which was thus chosen to be the study subject. JMJD6 not only catalyzes the demethylation of H3R2me2 and/or H4R3me2 but also impacts a variety of non-histone-modified factors [29]. In addition, JMJD6 is also a histone targeting lysine hydroxylase, which results in lysine hydroxylation on distinct protein substrates for the regulation of gene transcription [30]. The overexpression of JMJD6 has been validated in different cancers, including neuroblastoma and melanoma [31, 32]. After determining the interaction between JMJD6 and CCNB2, we explored the effects of their binding relation on the p53 pathway. Consistent with our prediction results, CCNB2 knockdown activated the p53 pathway in NPC cells, which was mitigated by JMJD6, preliminarily confirming the JMJD6/CCNB2/p53 axis in NPC. To further corroborate the function of this axis, NPC cells transfected with si CCNB2 were further transfected with JMJD6 OE or p53 inhibitor PFTα. JMJD6 OE or PFTα treatment mitigated the anti-tumor effects of si CCNB2 both in vitro and in vivo. JMJD5 was exhibited to be vital for sustaining cell migration and invasion, and the depletion of JMJD5 increased E-cadherin expression patterns and diminished N-cadherin and Vimentin expression in oral squamous cell carcinoma cells [33]. Similarly, JMJD6 overexpression facilitated EMT in the present study, as evidenced by lowered E-cadherin and ZO-1, and enhanced N-cadherin and Vimentin expression. Also, the regulatory effects of JMJD6 on the cell cycle of breast cancer cells were verified as well [34]. JMJD6 inhibition has been revealed to suppress proliferation and induce apoptosis in ovarian cancer cells [35]. In addition, shRNA lentiviral vector-controlled depletion of JMJD6 in glioma stem cells contributed to decreased proliferation, migration, and invasion, and the underlying mechanism is related to the strengthening of the p53 signaling pathway [36]. JMJD6 promoted colon carcinogenesis through the regulation of p53 by hydroxylation [37]. Even though p53 has been revealed to inhibit CCNB1 and CCNB2 in hepatocellular carcinoma [38], there is also evidence suggesting CCNB1 downregulated p53 expression by promoting its proteasome degradation in hepatocellular carcinoma [39, 40]. The inhibitory effect of CCNB2 on p53 signaling was also observed in our experiments. Future studies will be needed to determine the molecular mechanisms governing the impairment of p53 by CCNB2.

## Conclusion

**Fig. 6:**
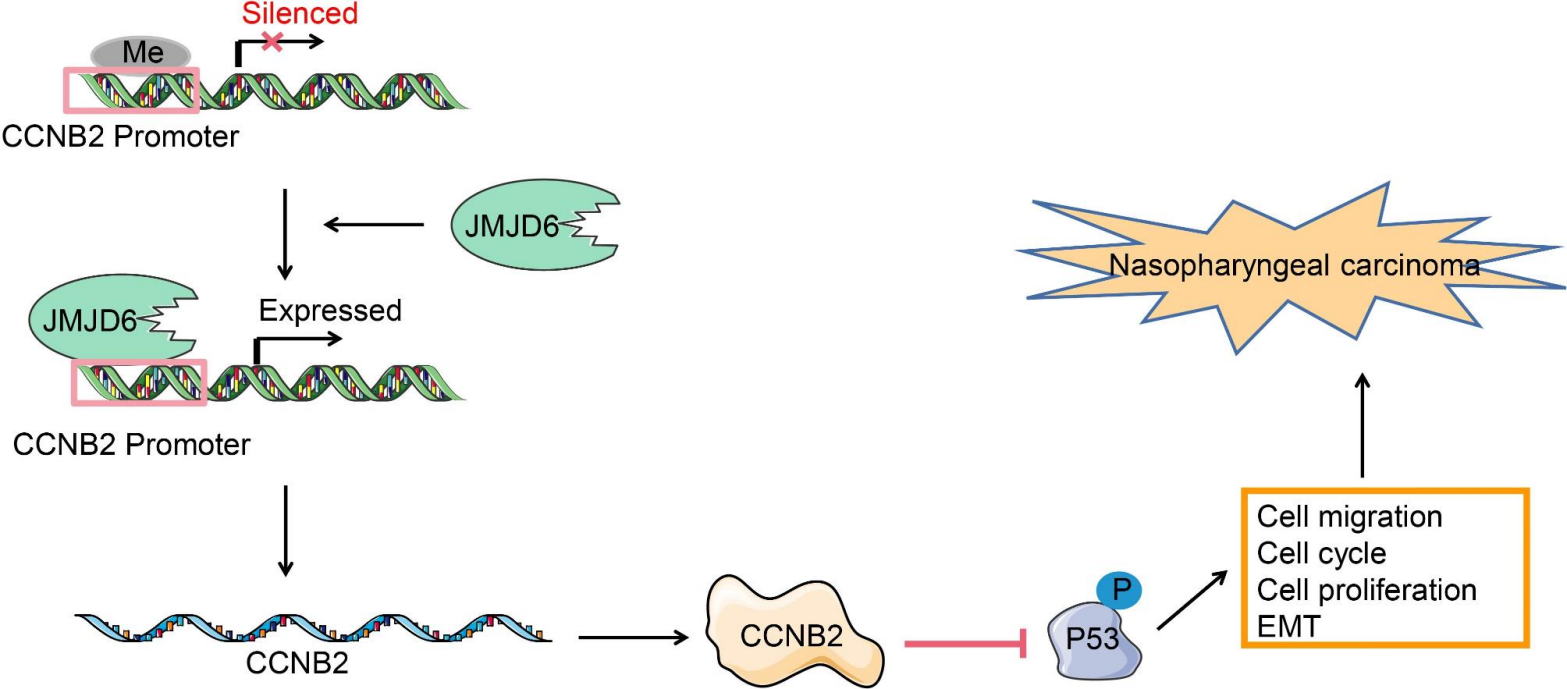
A proposed model of molecular interaction to delineate the action mechanisms of CCNB2 in NPC. JMJD6 is highly expressed in NPC and upregulates CCNB2 expression by binding to the CCNB2 promoter, which inhibits the P53 pathway and ultimately promotes the development of NPC.

Lastly, to generalize our findings, we recapitulated a positive relation between JMJD6 and CCNB2. Furthermore, JMJD6 binds to CCNB2 and negatively modulates the EMT of NPC cells via the p53 signaling termination, eventually promoting tumor growth and metastasis (Fig. 6). CCNB2 inhibition may serve as a potential molecular therapeutic target against NPC.

### Electronic supplementary material

Below is the link to the electronic supplementary material.

**Additional file 1**: Additional Figures.

**Additional file 2**: Additional Tables.

**Additional file 3**: Original Data.

## Data Availability

The analyzed data sets generated during the study are available from the corresponding author upon reasonable request.

## Abbreviations

NPC: Nasopharyngeal carcinoma
DMG: Differentially methylated genes
DEG: Differentially expressed genes
CCNB2: Cyclin B2
EMT: Epithelial-mesenchymal transition
GO: Gene Ontology
KEGG: Kyoto Encyclopedia of Genes and Genomes
RPMI: Roswell Park Memorial Institute
FACS: Fluorescence-activated cell sorting
PI: Propidium iodide
qMSP: Quantitative methylation-specific PCR
