# Supplementary material for: Cyclin B2 impairs the p53 signaling in nasopharyngeal carcinoma
Source: BMC Cancer. 2024 Jan 2;24:25. doi: 10.1186/s12885-023-11768-4 (PMC10763327; doi:10.1186/s12885-023-11768-4)
Supplement: Supplementary file 1 — Additional file 1: Additional Figures. [file 12885_2023_11768_MOESM1_ESM.docx]

**Supplementary Figure S1**

**
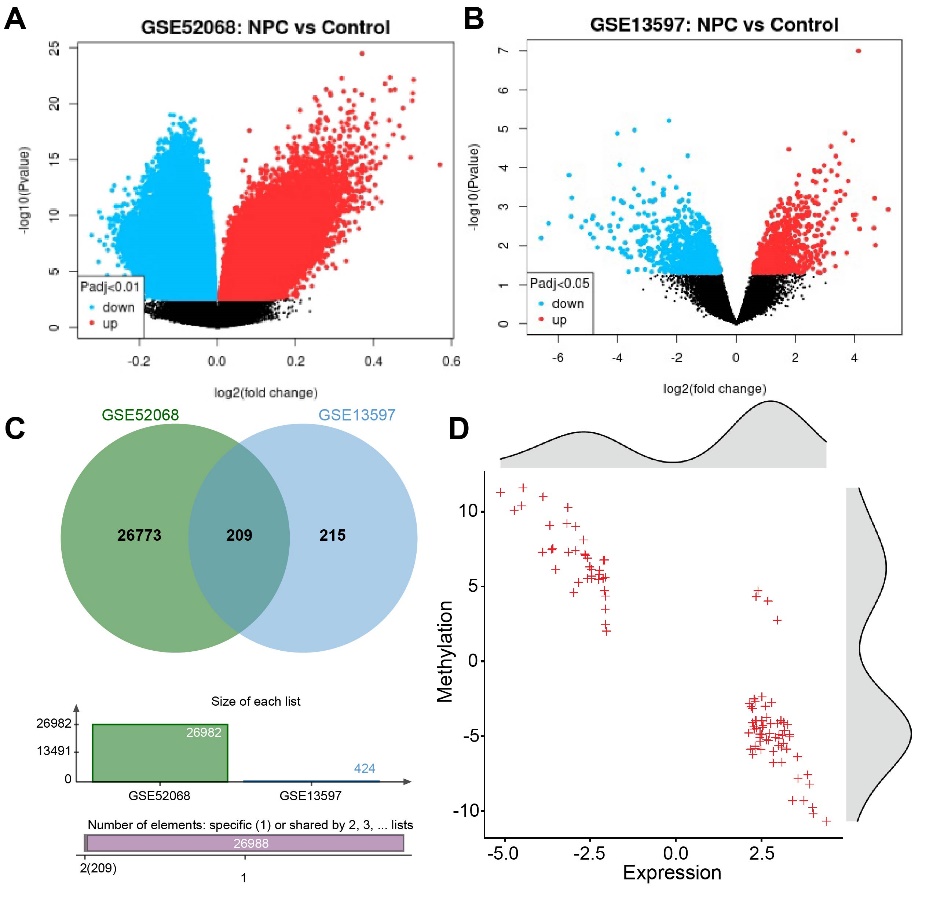
**

**Supplementary Figure S1** DMG and DEG in NPC. A, The GEO dataset GSE52068 was selected to analyze DMG in NPC, with *p* < 0.01 as the significance threshold. B, The GEO dataset GSE13597 was selected to analyze DEG in NPC with *p* < 0.01, |logFC| > 1.5 as the significance threshold. C, Overlapping of DMG and DEG using a Venn map to obtain 209 target genes. D, Scatter plot with gene density distribution to assess the reliability of overlapping genes where the horizontal coordinate represents the expression of genes in NPC, and the vertical coordinate represents the methylation level of genes.

**Supplementary Figure S2**

**
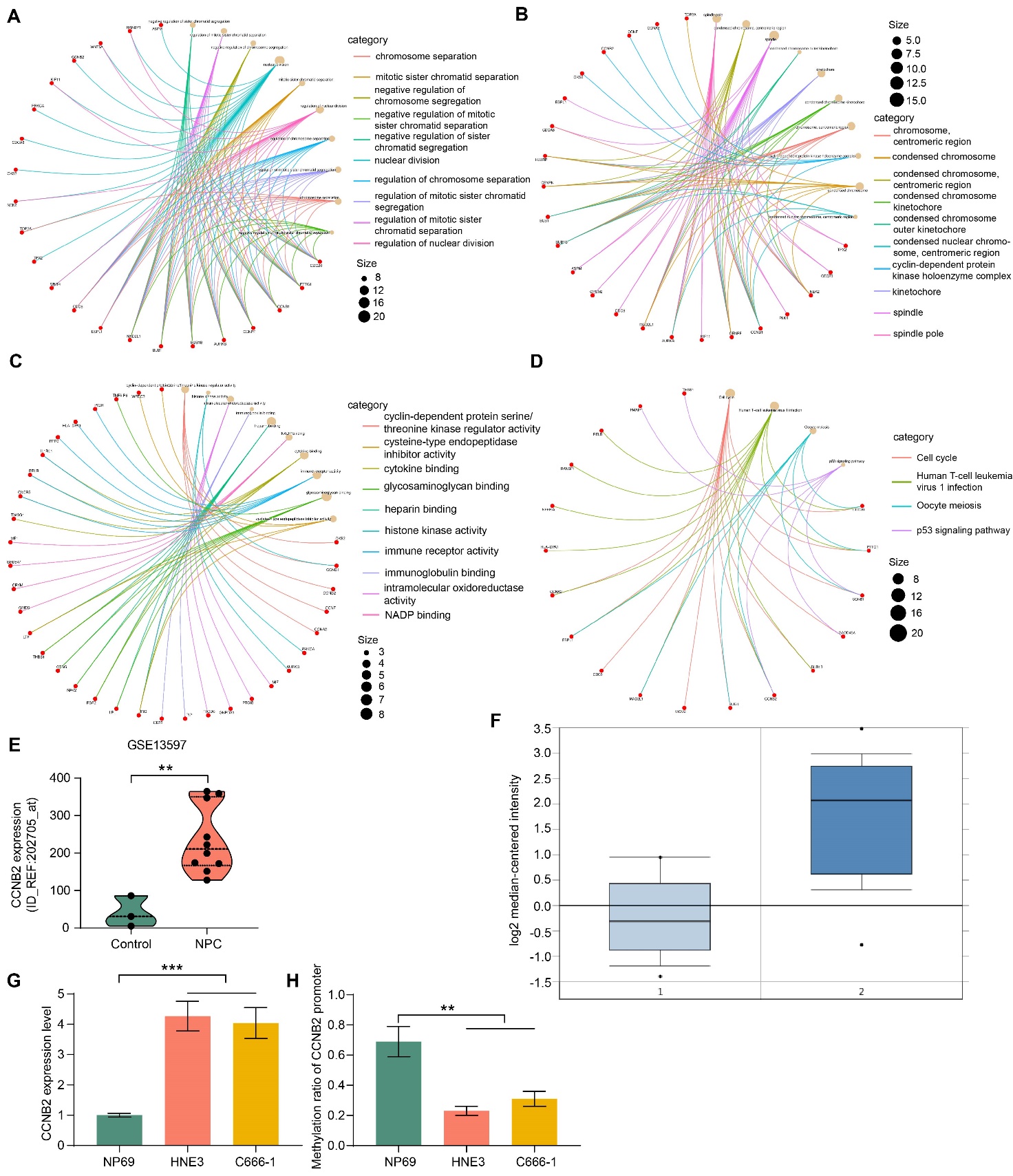
**

**Supplementary Figure S2** CCNB2 is both a DMG and DEG in NPC. A, Biologic process regulated by 209 intersecting genes using GO annotation. B, Cellular component of 209 intersecting genes using GO annotation. C, The molecular function exerted by 209 intersecting genes using GO annotation. D, KEGG pathway enrichment analyses of 209 intersecting genes. E, CCNB2 expression in NPC samples (n = 10) and control samples (n = 3) from GEO dataset GSE13597 (analyzed by the Mann Whitney test, ** represents *p* < 0.01). F, CCNB2 expression was analyzed in the Oncomine database, where 1 is normal nasal tissue and 2 denotes NPC tissue (*p-value* = 4.30E-5, FC value = 2.440). G, RT-qPCR was performed to detect the expression of CCNB2 in NPC cell lines HNE3 and C666-1 compared to normal human nasopharyngeal epithelial cell line NP69 based on the threshold cycle value (Ct) of qPCR using the 2^-ΔΔCt^ calculation method, and NP69 was used as a control group (n = 3, analyzed by the one-way ANOVA, ***represents *p* < 0.001). H, The methylation levels of CCNB2 promoter in NPC cell lines HNE3 and C666-1 compared to normal human nasopharyngeal epithelial cell line NP69 using qMSP, and the threshold cycle value of methylated qPCR/threshold cycle value of non-methylated qPCR was used to calculate the methylation level of CCNB2 promoter in each group of cells (n = 3, analyzed by the one-way ANOVA, **represents *p* < 0.01). Data are represented as mean ± SD.
