# Supplementary material for: Cyclin B2 impairs the p53 signaling in nasopharyngeal carcinoma
Source: BMC Cancer. 2024 Jan 2;24:25. doi: 10.1186/s12885-023-11768-4 (PMC10763327; doi:10.1186/s12885-023-11768-4)
Supplement: Supplementary file 2 — Additional file 2: Additional Tables. [file 12885_2023_11768_MOESM2_ESM.docx]

**Supplementary Table 1** 209 methylation-affected DEG

| ATP2C2 | CCNB1 | CYP2F1 | SGSM3 | LLGL1 | IL1RL1 |
| --- | --- | --- | --- | --- | --- |
| VPS45 | GADD45A | RALA | PMAIP1 | MPHOSPH8 | TUBA4B |
| MICAL2 | FPR3 | GNPDA1 | CHST3 | C7 | SCGB1A1 |
| WFDC2 | CDCA3 | LDLRAP1 | EPAS1 | DHX34 | TTTY14 |
| MAN1C1 | ARNT2 | SNX10 | APOC1 | INTS7 | TNFAIP6 |
| LPL | ALDH3B1 | PTGER1 | TNFAIP3 | MMP10 | HOXA10 |
| GDF7 | FOXJ1 | IL23A | ERN2 | SELENBP1 | ACSM5 |
| FERMT1 | CENPF | MAP3K13 | CPSF6 | DST | LTF |
| WNT4 | NOL10 | KIF11 | CTSG | TF | URB1 |
| HDGFRP3 | MYO15B | SERTAD2 | CLU | DNAI1 | HRG |
| PTGS2 | CDCA8 | CRYM | ZNF124 | H2AFX | GMNN |
| GJA4 | NBEA | CHN2 | CLCN4 | BPESC1 | C11orf63 |
| AGMAT | ADAM19 | TTC9 | MTA2 | RANBP1 | RFC3 |
| BMP2 | UMPS | AURKB | PER2 | FAM149A | NUDT13 |
| MECOM | SLC22A3 | COL14A1 | NFKBIA | CDC6 |  |
| SLC34A2 | HLF | BUB1B | ZBTB20 | RELB |  |
| KIF13B | IGF2BP3 | SLC44A4 | LMNB2 | CENPN |  |
| NUCB2 | POU2F2 | MUC5B | SEZ6L | ESPL1 |  |
| TPX2 | NPL | SGK1 | CD22 | GPSM2 |  |
| CNTNAP2 | ZNF639 | CXCR5 | NPTX1 | HJURP |  |
| AQP5 | WASF3 | GNG13 | OR3A2 | PSMD5 |  |
| MIF | PIP | CCNB2 | MCM2 | RCN2 |  |
| FGF2 | KIF3C | AZGP1 | TMEM63A | NUP214 |  |
| TOP2A | MGAT4C | BAIAP3 | VIPR1 | ASPM |  |
| OSBPL10 | GNMT | CFB | SOX4 | AGFG2 |  |
| PBK | STK19 | GATA2 | ST8SIA3 | RUNX1T1 |  |
| CDC20 | CD70 | NOX4 | MAD2L1 | STMN1 |  |
| TRIM31 | TCEAL2 | HLA-DPA1 | NIPAL3 | ELF5 |  |
| PTGIS | PRKCA | PKNOX1 | MIA3 | CCNA2 |  |
| NEK2 | SLPI | CHAF1B | SRD5A1 | THBS1 |  |
| MKRN2 | SECISBP2L | GPD1L | CCNF | TFF3 |  |
| PTTG1 | CNIH3 | PER3 | CCDC30 | ASXL3 |  |
| TK2 | TSPAN1 | ALDH1L1 | GNAL | TK1 |  |
| SNTG2 | LHX1 | ADRA2A | PIGR | GALNT3 |  |
| FSCN1 | SOBP | BUB1 | PRB4 | ME1 |  |
| GMDS | DNALI1 | PRB3 | ANK2 | DOPEY1 |  |
| CKS2 | NAV2 | NUP54 | SORBS2 | PRLR |  |
| PLK4 | KRT15 | MMD | WNT5A | EDNRB |  |
| TWSG1 | KRT32 | PTGDS | DTL | C4orf19 |  |
