# Supplementary figures and images for: Cyclin B2 impairs the p53 signaling in nasopharyngeal carcinoma
Source: BMC Cancer. 2024 Jan 2;24:25. doi: 10.1186/s12885-023-11768-4 (PMC10763327; doi:10.1186/s12885-023-11768-4)

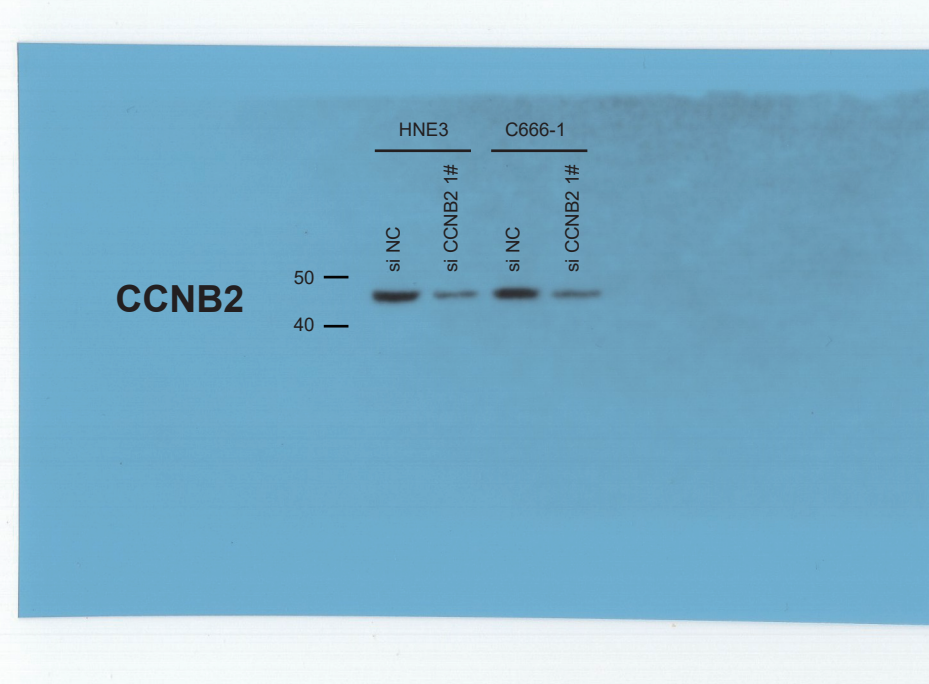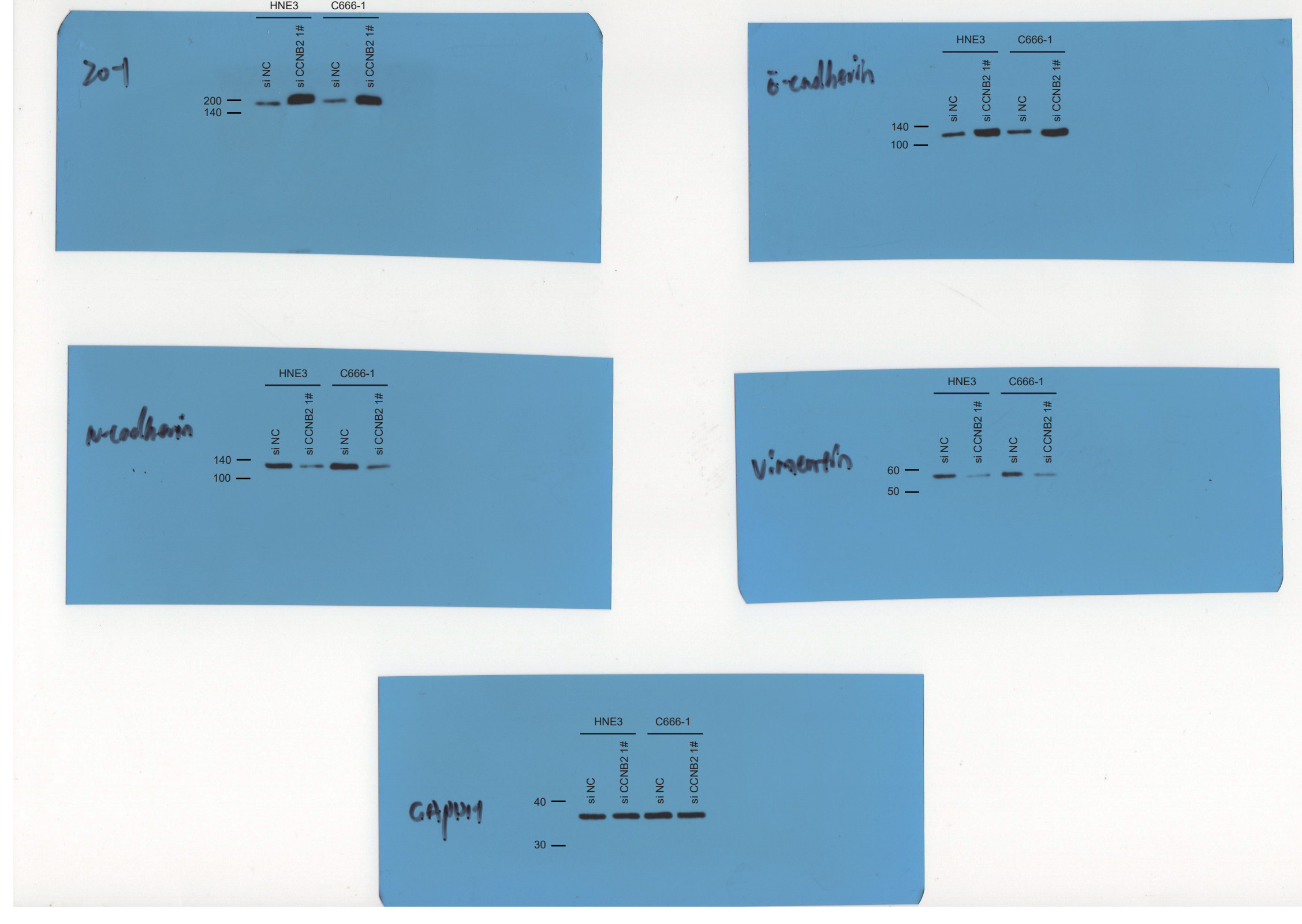

Fig 2F

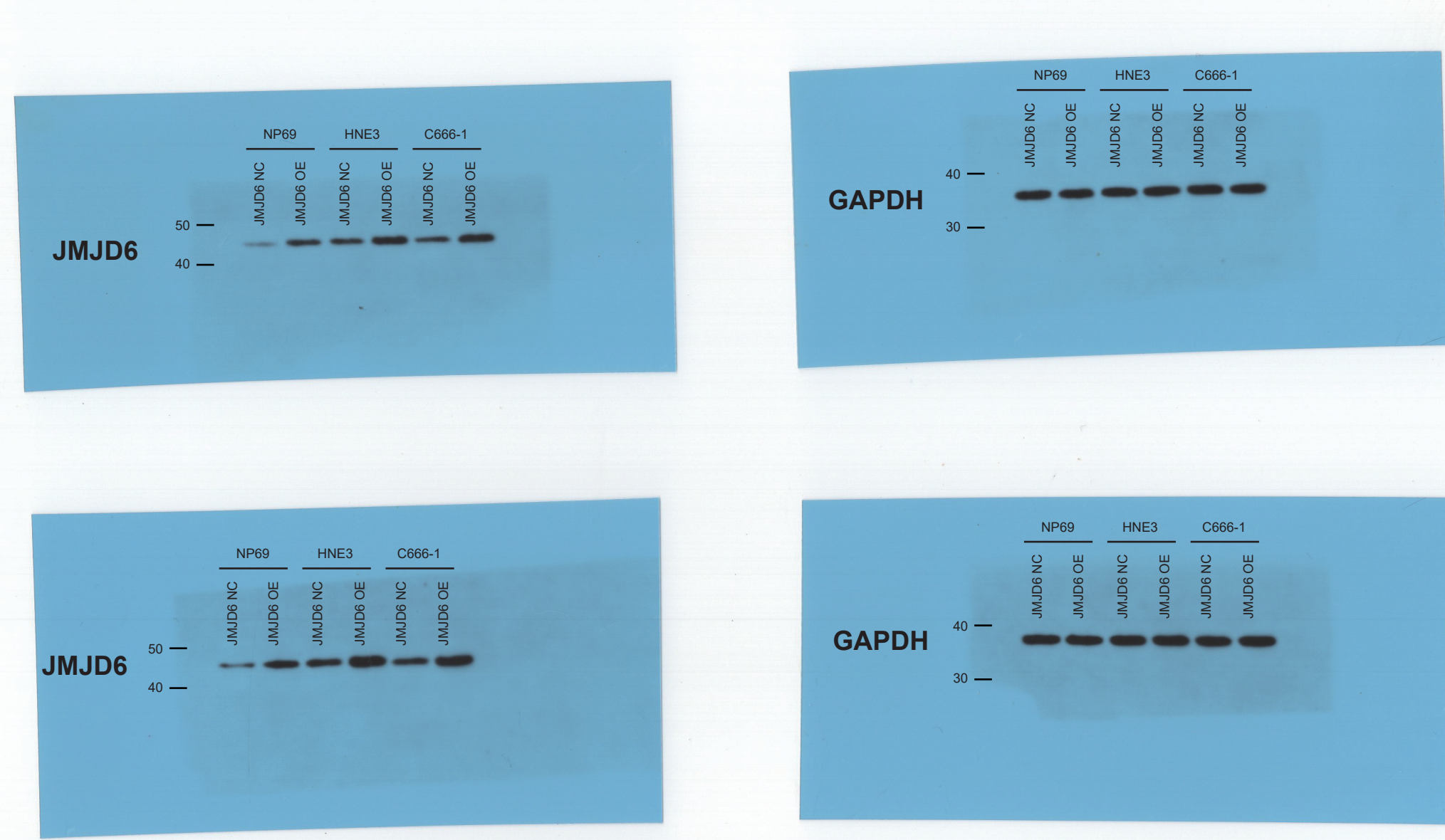

Fig 3A

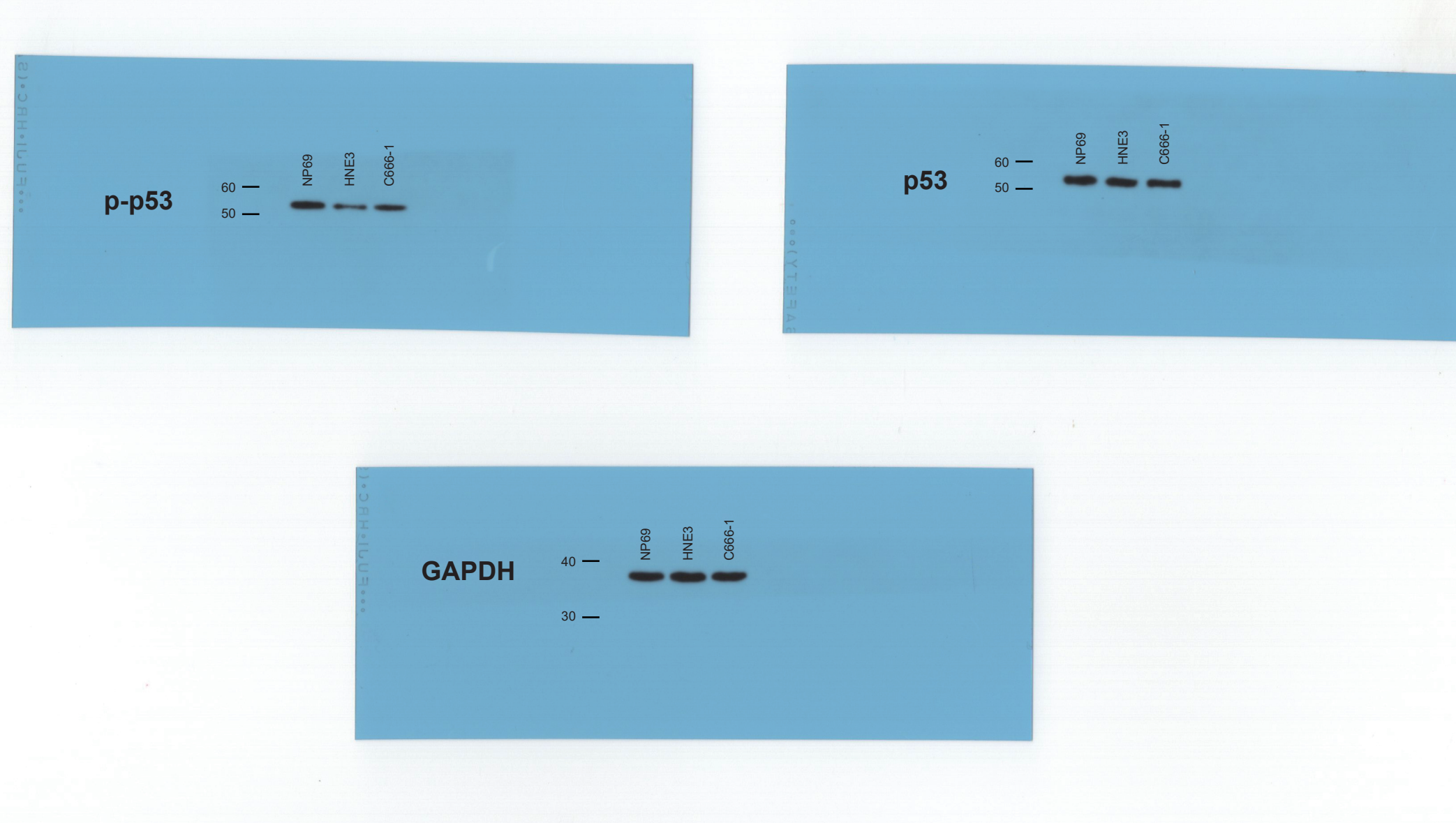

Fig 3B

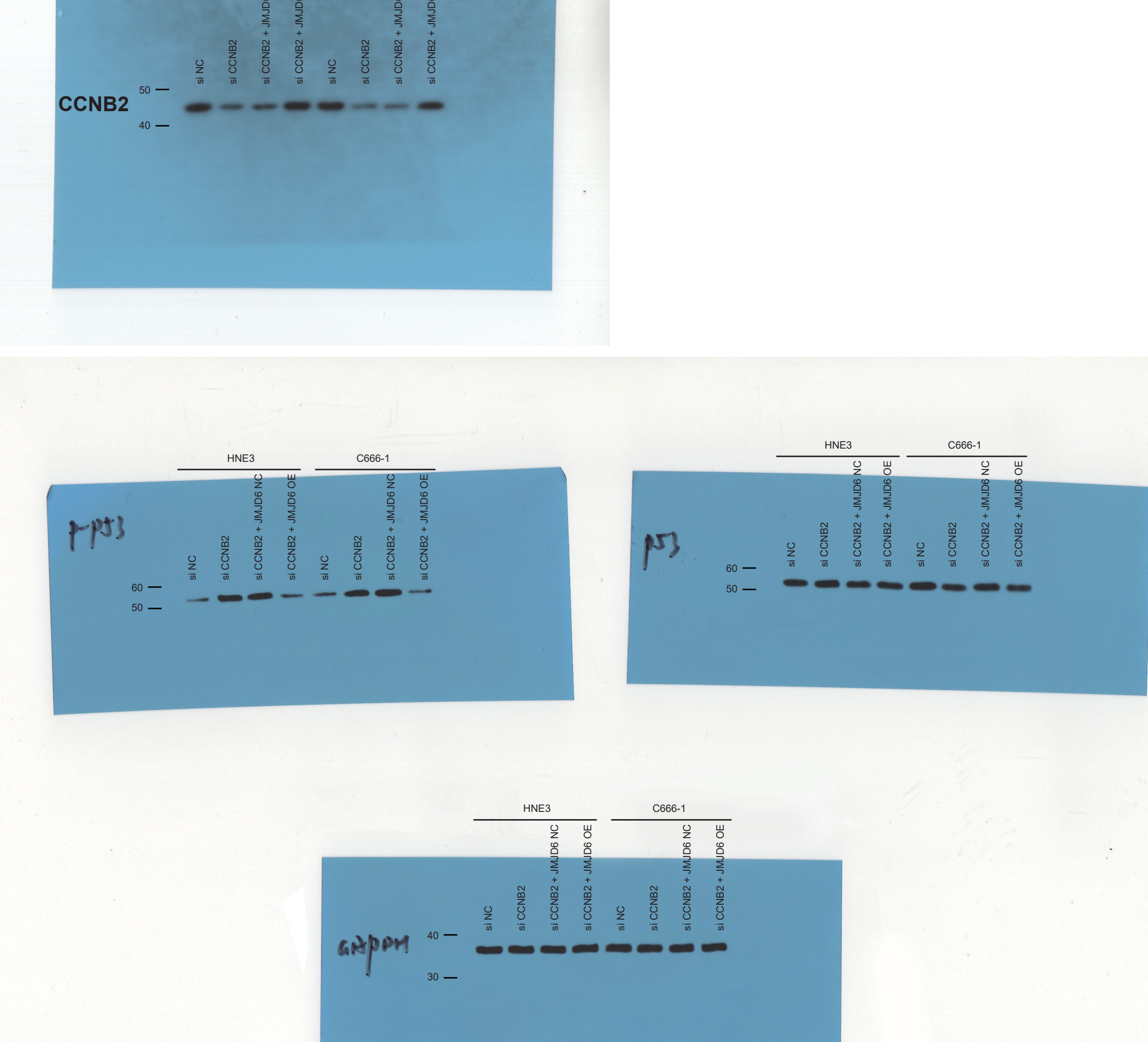

Fig 4A

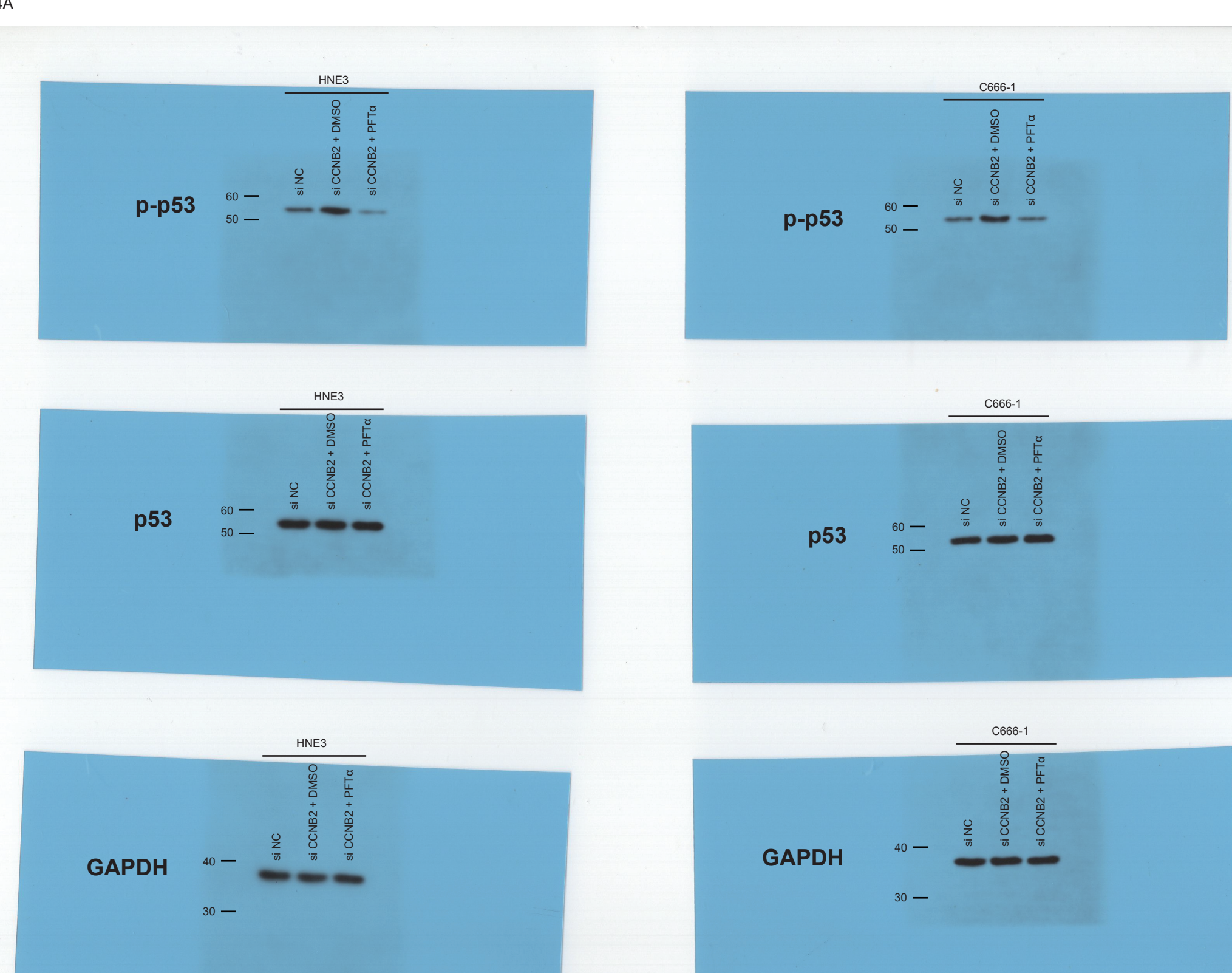

Fig 4G

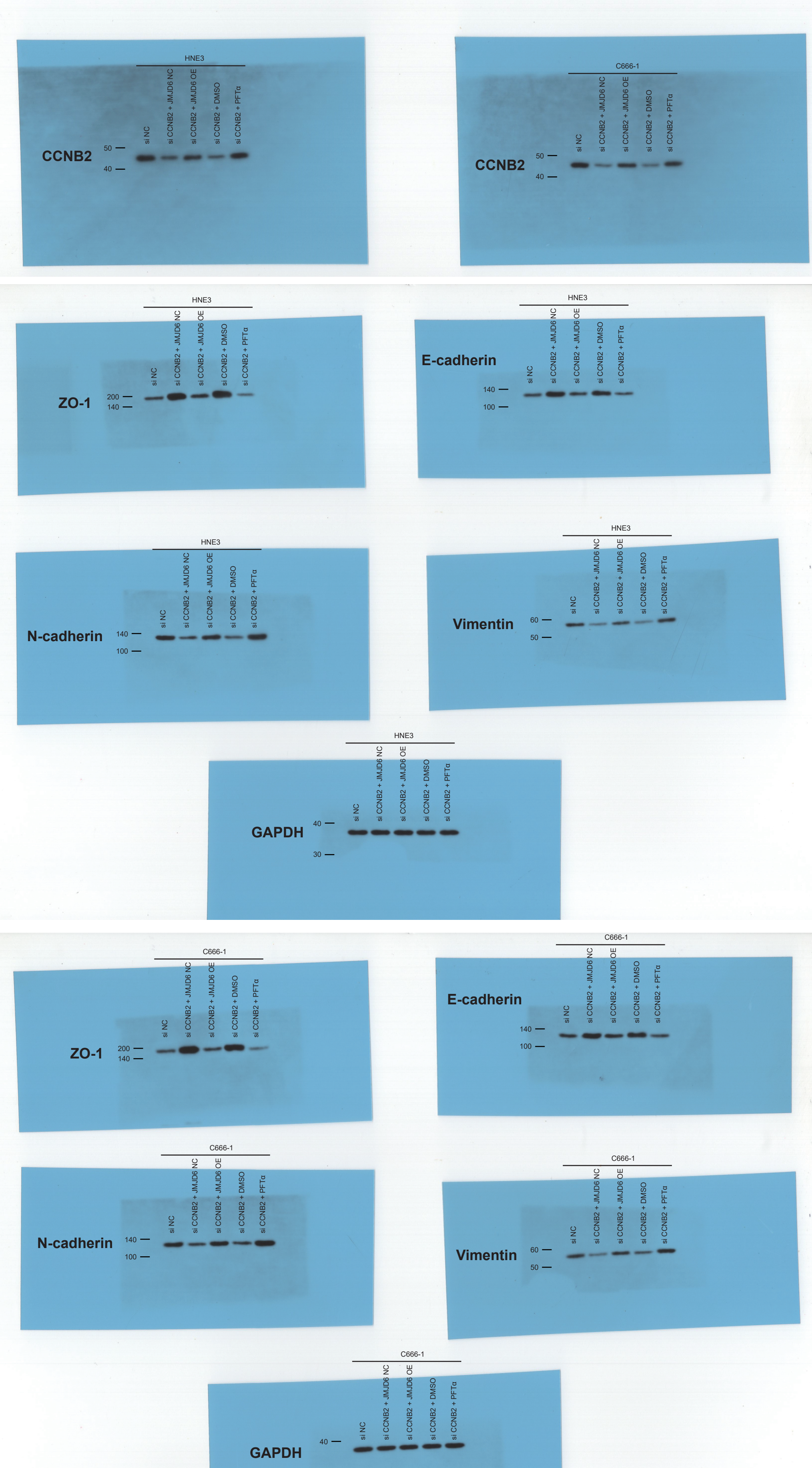

Supplement: Supplementary file 3 — Additional file 3: Original Data. [file 12885_2023_11768_MOESM3_ESM.pdf]
